# Assessment of self-medication practices and its associated factors among undergraduate students of private universities in Banadir region, Somalia

**DOI:** 10.1186/s12909-026-08818-3

**Published:** 2026-02-19

**Authors:** Samira Abdullahi Moalim, Hassan Mohamud Dirie, Jamal Hassan Mohamoud, Khadra Mohamed Hashi, Farhio Abdullahi Ali, Khawla Abdirahman Abubakar

**Affiliations:** 1 Department of Microbiology and Laboratory Sciences, Faculty of Medicine and Health Sciences, SIMAD University, Mogadishu, Somalia; 2 Department of Public Health, Faculty of Medicine and Health Sciences, SIMAD University, Mogadishu, Somalia; 3 SIMAD Institute for Global Health (SIGHt), SIMAD University, Mogadishu, Somalia

**Keywords:** Self-medication, Undergraduate students, Somalia

## Abstract

**Background:**

Self-medication involves taking drugs or herbal products to treat self-diagnosed symptoms or health conditions without consulting a healthcare professional. It is a common practice and an important component of day-to-day healthcare management, particularly in developing countries, where access to health services may be limited. Health science students are often believed to be self-medicated more frequently than the general population due to their medical knowledge. This study aimed to determine the prevalence of self-medication and associated factors among undergraduate students in private universities in the Banadir region of Somalia.

**Methods:**

An institution-based cross-sectional study was conducted among 679 undergraduate students from private universities in the Banadir region. Participants were recruited using a convenience sampling technique. Data were collected using a self-administered questionnaire and analyzed using descriptive statistics and logistic regression to identify factors associated with self- medication.

**Results:**

The study found that 67% of students practiced self-medication. Analgesics (50%) and antibiotics (38%) were the most used medications, primarily for headache (33.1%) and fever (18.7%). Students without prior training in antibiotic use were significantly more likely to practice self- medication, as were non-medical students compared with students enrolled in medical fields.

**Conclusion:**

Self-medication was highly prevalent among Somali undergraduates, with frequent use of analgesics and antibiotics, particularly among non-medical students and those without prior training on antibiotic use. These practices pose risks, including adverse drug effects, delayed diagnosis, and contribution to antimicrobial resistance. Targeted educational interventions strengthened training on rational antibiotic use across university programs, and stricter enforcement of prescription-only regulations are recommended to promote safer medicine use.

## Background

Self-medication refers to the use of medications to treat illnesses or symptoms that individuals identify themselves [1]. It is characterized as the ingestion of medication without a prescription, regardless of the reason, dosage, or duration of use [2]. Inappropriate self-medication, particularly with antibiotics, poses serious risks to individual and public health and is a major driver of antimicrobial resistance worldwide [3]. Self-medication with antibiotics (SMA), defined as the use of antibiotics for the personal treatment of ailments or symptoms without a prescription or professional guidance, represents a significant global health concern. However, estimating the true prevalence of SMA remains challenging due to the reliance on self-reported questionnaires in studies conducted across different countries [4].

Numerous studies indicate that young adults are particularly susceptible to self-medication due to factors such as reduced risk perception regarding drug use, partial drug knowledge, easy access to the internet, extensive media coverage of health-related topics, widespread availability of medicines, educational attainment, and social influences. Self-medication has been widely investigated across various populations in Africa, Asia, and Europe [5]. University students are considered a high-risk group for self-medication because of increased autonomy, partial medical knowledge, and easy access to medicines [6]. The global rise in self-medication has been driven by economic, political, and cultural factors, making it an important public health issue. Nevertheless, substantial differences exist between developing and developed countries, largely due to variations in socioeconomic conditions, cultural practices, and healthcare system characteristics, including access to care, reimbursement policies, and medication dispensing regulations [7].

In many low- and middle-income countries, inadequate regulation of medicine sales enables easy access to prescription-only drugs, thereby promoting self-medication practices [8]. Educational attainment is among the key factors influencing self-medication behavior. Although self- medication among tertiary students has been extensively studied worldwide, no comparable study has been conducted in Somalia. Investigating self-medication among university students is particularly important, as this group represents a highly educated and health-aware segment of society. Moreover, studying medical and clinical students—who will become future prescribers and health educators—is especially critical [9].

Previous studies have identified several common reasons for self-medication, including prior experience with similar illnesses, cost and time efficiency, minor health conditions, the need for immediate symptom relief, recommendations from family or friends, convenience, ease of drug access, dissatisfaction with healthcare services, and unfriendly behavior of healthcare professionals. Cough and headache have been reported as the most common conditions prompting self-medication, followed by fever and the common cold [10].

Beyond its widespread prevalence, self-medication carries serious consequences for both individuals and health systems. Clinically, it increases the risk of misdiagnosis or delayed diagnosis, adverse drug reactions, harmful drug interactions, and inappropriate selection or dosing of treatment [11]. Inappropriate self-medication with antibiotics has been associated with treatment failure, masking of serious illnesses, and an increased risk of drug-resistant infections, which complicate clinical management and prolong disease duration [12]. From an economic perspective, although self-medication may appear cost-saving in the short term, antimicrobial resistance and treatment failures can lead to substantial costs through prolonged hospital stays, additional diagnostic testing, and the use of more expensive therapies [13]. Despite extensive documentation of antibiotic self-medication globally, evidence remains limited in low-resource and conflict-affected settings, constraining the development of effective, context-specific interventions [14]. Of particular concern is the contribution of unsupervised antibiotic use to antimicrobial resistance, which represents one of the most pressing global health threats and imposes a significant burden on healthcare systems worldwide [15].

The findings of this study will contribute to a better understanding of self-medication practices and support the development of public health strategies tailored to Somali undergraduates. Based on a review of the existing literature, this is the first study to examine self-medication among university students in Somalia. The aim of the study was to investigate the prevalence, patterns, and determinants of self-medication practices in this population, thereby providing evidence that can guide safer and more responsible medicine use.

## Methods and materials

### Study design and setting

Undergraduate students from the Banadir region of Somalia participated in a cross-sectional, institution-based study conducted between 30 May 2024 and 21 May 2025. Banadir, located in the southeastern part of Somalia, encompasses Mogadishu, the capital city and the country’s political, economic, and educational center. The region hosts several prominent private universities, including SIMAD University, Mogadishu University, the University of Somalia, and Jamhuuriya University of Technology and Sciences. These institutions were selected due to their large student enrollments and diverse academic programs, making them representative of the broader undergraduate student population in the region.

### Study population and eligibility criteria

The source population comprised all undergraduate students in the Banadir region, while the study population included undergraduates enrolled in the four selected private universities (SIMAD University, Mogadishu University, the University of Somalia, and Jamhuuriya University of Technology and Sciences). The study subjects were students who met the eligibility criteria and consented to participate.

Inclusion criteria consisted of students aged 18 years and above who were actively enrolled at the time of data collection. Students were informed about the study objectives and procedures through classroom announcements and official student WhatsApp groups. Participation was entirely voluntary, and students were free to decline participation without any penalty. Students who declined participation or were unavailable during the data collection period were excluded from the study.

### Sample size and sampling technique

The sample size was calculated using the single population proportion formula: $\mathrm{n}=\left(\mathrm{Z}^{2}\times\mathrm{p}\left(1-\mathrm{p}\right)\right)/\mathrm{d}^{2}$,

where *n* is the required sample size, *Z* is the Z-statistic at the 95% confidence level (1.96), *p* is the estimated prevalence of self-medication (0.80) obtained from a previous study, and *d* is the margin of error (0.03). This approach is recommended for prevalence surveys in health research [16].

Based on these parameters, the calculated sample size was 682 students. Three responses were incomplete and excluded from the final analysis. The sample was proportionally allocated across the selected universities according to their current student enrolment figures. A convenience sampling technique was employed to recruit participants.

### Data collection procedure

Data were collected using a self-administered questionnaire distributed through the Kobo Toolbox online platform. The questionnaire framework was reviewed against previously validated instruments to ensure relevance and appropriateness. The tool was adapted from validated and published questionnaires used in similar studies [1, 2, 7] with modifications made to enhance clarity, cultural appropriateness, and relevance to the study population.

The questionnaire was initially developed in English, translated into Somali, and subsequently back translated into English to ensure linguistic accuracy and cultural equivalence, in line with best practices.

The instrument consisted of four main domains:

1. Socio-demographic characteristics: age, sex, marital status, year and field of study, and monthly income.
2. Knowledge and training on antibiotic use: prior exposure to formal training or educational sessions related to antibiotics.
3. Self-medication practices: types of drugs used (e.g., analgesics, antibiotics), symptoms prompting self-medication, sources of medicines, and experienced adverse effects.
4. Reasons for practicing self-medication: predefined options including perceived minor illness, cost-saving, time constraints, prior experience, and mistrust of healthcare services.

For this study, “training” was broadly defined as any structured lecture, workshop, or formal educational session provided by the university or external health organizations, regardless of duration, provided it explicitly focused on antibiotic use and its appropriate application.

The structured, closed-ended design of the questionnaire facilitated systematic quantification of behaviours and motivations.

A pilot study was conducted at SIMAD University involving 34 students (approximately 5% of the total sample) to assess clarity, cultural appropriateness, and reliability of the instrument. Feedback from the pilot testing was used to revise ambiguous items, serving as a revalidation step. The questionnaire demonstrated good internal consistency, with a Cronbach’s alpha coefficient of 0.83.

### Data analysis procedures

Data were analysed using logistic regression techniques. Bivariate logistic regression was initially performed to examine crude associations between each independent variable and self-medication practice. Variables with a *p*-value less than 0.20 were included in the multivariable logistic regression model.

The final model adjusted for sex, age group, year of study, marital status, and monthly income. Variables with a *p*-value less than 0.05 were considered statistically significant. Results were reported as adjusted odds ratios (AORs) with 95% confidence intervals (CIs). Model fitness was assessed using the Hosmer–Lemeshow goodness-of-fit test.

### Ethical approval and informed consent

Ethical approval for the study was obtained from the Institutional Review Board (IRB) of the Faculty of Medicine and Health Sciences, SIMAD University, Mogadishu, Somalia (Ref#: 2025/SU-IRB/FMHS/P0024). Informed consent was obtained from all participants after a clear explanation of the study objectives and procedures was provided. Participation was voluntary, and respondents were informed of their right to withdraw at any stage without consequences. To maintain confidentiality, no personal identifiers were collected during the data collection process.

## Results

### Students’ characteristics

Of the 682 students invited to participate, data from three students were incomplete and therefore excluded, yielding a final sample of 679 students and a response rate of 99.6%. The demographic analysis showed that 49.8% of participants were younger than 20 years, 44.6% were aged 20–25 years, and 5.6% were older than 26 years. Females constituted most participants (58.0%), compared to males (42.0%). Regarding marital status, 94.0% were single, while 6.0% were married. Most students were enrolled in medical-related undergraduate programs (66.5%), whereas 33.5% were enrolled in non-medical programs. Additionally, 44.9% of students reported having received training on antibiotic use, while 55.1% reported no such training (Table 1).

**Table 1:** Students’ characteristics

Parameters	*N*	%
**Age**		
< 20 years	338	49.8
20–25 years	303	44.6
> 26 years	38	5.6
**Gender**		
Male	285	42.0
Female	394	58.0
**Marital status**		
Married	41	6.0
Unmarried	638	94.0
**Field of study**		
Non-Medical	222	33.5
Medical	44	66.5
**Training in antibiotic use**		
No	374	55.1
Yes	305	44.9

### Self-medication practices among undergraduate students

**Fig. 1:**
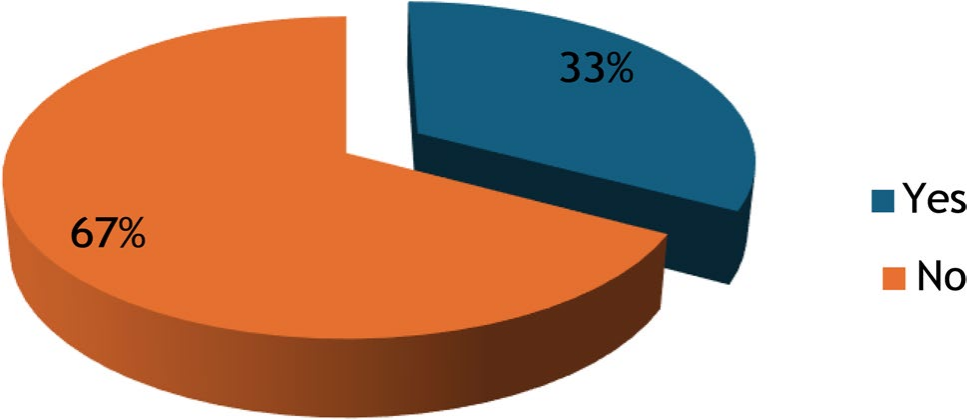
Prevalence of self-medication among undergraduate students in Banadir Region, Somalia

The prevalence of self-medication among undergraduate students was 67% (Fig. 1).

### Distribution of medications among students

The findings showed that analgesics were the most used medications (50%), followed by antibiotics (38%) and antimalarial drugs (10%). Among antibiotics, amoxicillin was the most frequently used (41.6%), followed by penicillin (20.0%), amoxiclav (15.5%), erythromycin (11.66%), and azithromycin (11.11%).The most common symptoms prompting self-medication were headache (33.1%), followed by fever (18.7%), cough (13.71%), gastrointestinal problems (12.71%), sore throat (10.97%), and other symptoms (10.72%). Most students obtained medications from pharmacies (56.08%), while previous prescriptions, friends or relatives, online sources, and other means accounted for the remaining sources. Reported adverse effects included allergic reactions (37.91%), antibiotic resistance (19.58%), digestive problems (12.0%), worsening of the original condition (8.75%), and other effects (21.66%) (Table 2).

**Table 2:** Distribution of medications among undergraduate students

Parameters	*N*	%
**Medications used in the Past Six Month**		
Analgesics	140	50
Antibiotics	105	38
Antimalarial drugs	30	10
**Types of Antibiotics**		
Amoxicillin	75	41.6
Amoclavin	28	15.5
Azithromycin	20	11.11
Penicillin	36	20
Erythromycin	21	11.66
**Symptoms**		
Headache	133	33.1
Fever	75	18.7
Cough	55	13.71
Sore throat	44	10.97
Gastrointestinal	51	12.71
Other	43	10.72
**Sources of Medications**		
Pharmacy	166	56.08
Previously Prescribed Medicine	53	17.90
Friends or Relatives	35	11.82
Online Sources	20	6.75
Others	22	7.43
**Adverse side effects of Medications**		
Allergic Reactions	91	37.91
Antibiotic Resistance	47	19.58
Digestive Problems	29	12.0
Worsening of the original condition	21	8.75
Other	52	21.66

### Reasons for practicing self-medication

**Fig. 2:**
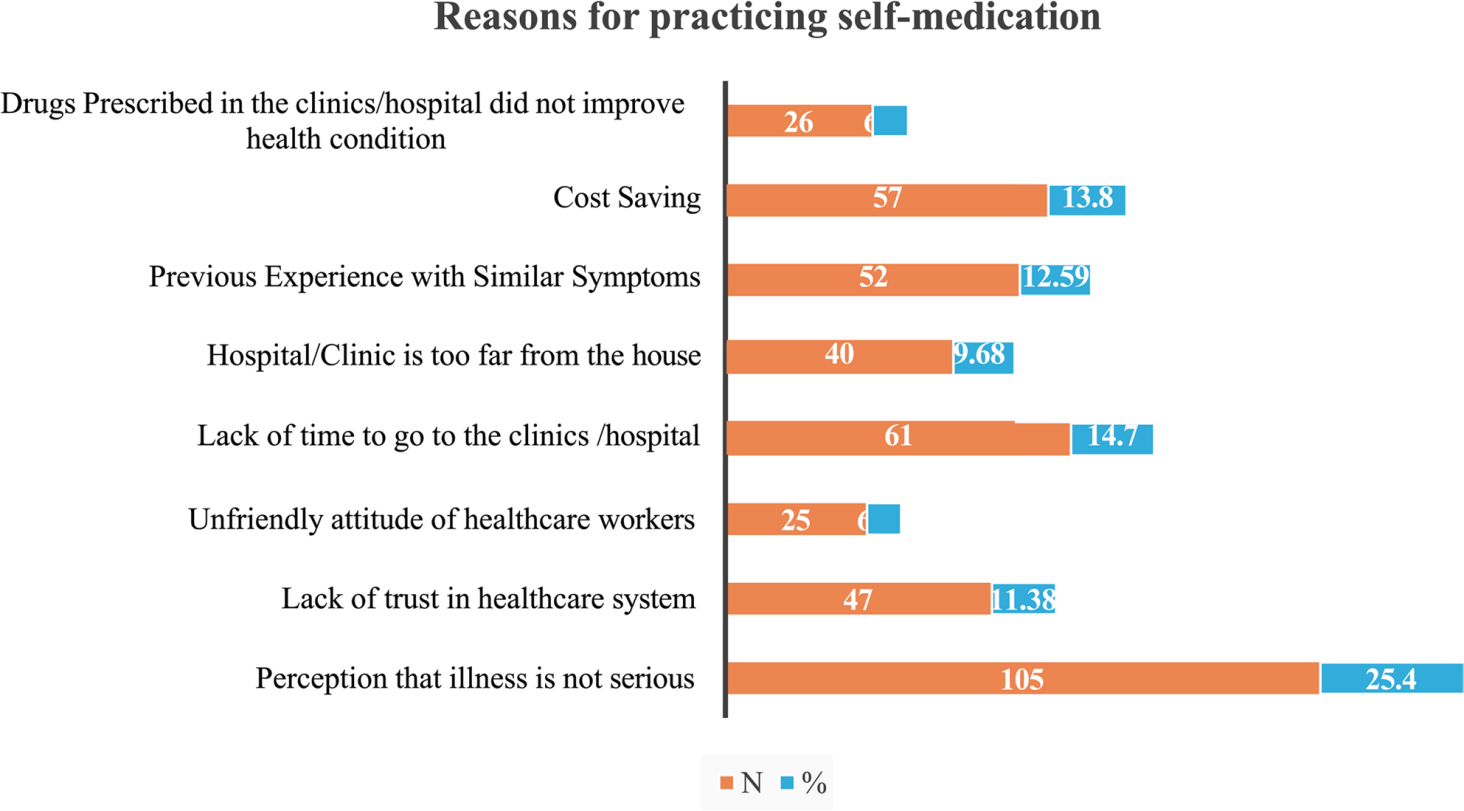
Illustrates the reasons for self-medication among undergraduate students in the Banadir region

**Table 3:** Associated factors with self-medication practices among undergraduate students in Banadir Regions, Somalia

Parameters	*N* %	Self-medication Practices		COR (95%)	*P* value	AOR (95%)	*P* value
		Yes	No				
**Age in Years**							
< 20 years	90 (40.4)	26 (41.9)	64 (39.8)	1			
20–25 years	120 (53.8)	35 (56.5)	85 (52.8)	0.98(0.54–1.80)	0.965		
> 26 years	13 (5.8)	1 (1.6)	12 (7.5)	4.87(0.60-39.42)	0.137		
**Gender**							
Male	285 (42.0)	94 (42.2)	191 (41.9)	1			
Female	394 (58.0)	129 (57.8)	265 (58.1)	1.01 (0.073-1.39)	0.947		
**Marital status**							
Married	41(6.0)	14(6.3)	27(5.9)	1			
Unmarried	638(94.0)	209(93.4)	429(94.1)	0.94(0.48–1.82)	0.855		
**Field of study**							
Non-Medical	222(33.5)	48(22.1)	174(39.0)	0.44(0.30–0.64)	< 0.0001*	1.62 (1.91–2.42)	0.017*
Medical	441(66.5)	169(77.9)	272(61.0)	1			
**Training on antibiotic use**							
No	374(55.1)	65(19.1)	309(67.8)	5.11(3.60–7.24)	< 0.0001*	4.73 (3.30–6.79)	< 0.0001*
Yes	305(44.9)	158(70)	147(32.2)	1			

Bivariate logistic regression was first used to examine crude associations between independent variables and self-medication. In this model, non-medical students initially appeared less likely to practice self-medication compared to medical students (COR = 0.44, *p* < 0.001). However, after adjusting for sex, age group, year of study, marital status, and monthly income in the multivariable logistic regression, the direction of association reversed, with non-medical students 1.62 times more likely to practice self-medication than medical students (AOR = 1.62, *p* < 0.001) (Table 3).

## Discussion

Self-medication is prevalent in both developed and developing nations [5]. Numerous studies have shown that self-medication practices are widespread, with variations observed globally [1]. This study aimed to evaluate self-medication practices and associated factors among undergraduate students in the Banadir region of Somalia. The findings indicate that the prevalence of self-medication among undergraduates in Banadir was 67%. Comparable results have been reported among university students in Uganda (63.5%). However, this prevalence exceeded that reported among university students in Gondar, Ethiopia (38.5%), and Mekelle, Ethiopia (43.24%) [10].

The high prevalence of self-medication may be attributed to the perception that illnesses are not serious (25.4%), lack of time to visit healthcare facilities (14.7%), and cost-saving considerations (13.8%). These findings may reflect the educational background and socio-economic characteristics of the participants.

The survey revealed that analgesics, such as paracetamol and ibuprofen (50%), and antibiotics (38%) were the most frequently used medications for self-medication. Similar patterns have been reported in studies conducted in Iran, Turkey, Saudi Arabia, Finland, and Spain, where analgesics and antibiotics were commonly used for self-medication [17]. A high prevalence of antibiotic self- medication has also been documented among health science students in Arsi, Ethiopia (58%) [10].

In these studies, analgesics were primarily used to relieve headache and musculoskeletal pain, while antibiotics were often self-medicated without prescription for respiratory and gastrointestinal infections, reflecting patterns like those observed in the present study.

Headache (33.1%) and fever (18.7%) were the predominant symptoms prompting self-medication, findings that are consistent with other studies. Headache is a common trigger for self-medication because it is associated with a wide range of conditions, leading individuals to seek immediate relief [18]. Studies in Ethiopia reported headache (32.1%) as the leading reason for self- medication, followed by stomach discomfort (22.6%) [5]. In addition to headache and fever, this study identified cough, sore throat, and gastrointestinal problems as additional reasons for self- medication, albeit at lower frequencies. Including these symptoms provides a more comprehensive understanding of the illnesses driving self-medication among undergraduate students.

The study identified significant associations between self-medication practices and certain demographic factors. Non-medical students were 1.62 times more likely to practice self- medication than medical students (AOR = 1.62, *p* = 0.017). The observed reversal in the direction of association after adjustment is likely attributable to confounding variables. In this study, medical students were disproportionately represented in senior academic years and higher income categories, which may have inflated their crude likelihood of self-medication. After controlling these variables in the multivariable model, the higher propensity for self-medication among non- medical students became evident. After controlling these variables in the multivariable model, the higher propensity of non-medical students became evident. This interpretation is supported by prior research, including an Ethiopian study that identified year of study and monthly income as predictors of self-medication [19] and a multicenter study from Saudi Arabia, which similarly found that senior students and higher-income groups were more likely to self-medicate [20]. In contrast, studies conducted in Iran reported no significant associations between self-medication and sex, marital status, or year of study [17] Where no significant association was found between sex, marital status, year of study [21] Such variation may reflect differences in health system regulation, cultural attitudes toward medicine use, and methodological differences in sampling and survey tools. Medical students may be more cautious in self-medication due to greater awareness of drug-related risks. Conversely, the increased likelihood of self-medication among non-medical students may stem from limited knowledge of medication risks, greater reliance on informal advice from peers or family, and the perception that self-medication is safe and convenient.

An additional explanation for the frequent use of antibiotics for self-medication in this study is the weak regulation of medicine distribution in Somalia. Although antibiotics are legally classified as prescription-only medications, regulatory enforcement remains limited, allowing students to purchase antibiotics directly from pharmacies or informal drug sellers without medical consultation. This finding aligns with reports highlighting Somalia’s largely unregulated antibiotic market [22]. A community-based study in Mogadishu similarly found that residents frequently obtain antibiotics from pharmacies based on perceived symptoms or non-medical advice, particularly in the context of financial constraints [23]. These regulatory gaps contribute to widespread misuse of antibiotics and emphasize the urgent need for stronger pharmaceutical regulation and pharmacy oversight to mitigate inappropriate access and reduce the risk of antimicrobial resistance.

Differences between the present findings and those reported in Gondar, Mekelle, and Iran may also be explained by contextual variations in cultural attitudes toward self-care, socioeconomic conditions, healthcare accessibility, and methodological differences in sampling and survey design. In Banadir, weak health infrastructure, widespread economic hardship, and reliance on informal advice networks may further exacerbate self-medication behaviors, particularly among non-medical students with limited awareness of associated risks.

The high prevalence of self-medication among undergraduates in Banadir (67%) has important public health implications. Such practices increase the risk of inappropriate drug use, adverse reactions, delayed diagnosis, and inappropriate treatment, potentially complicating disease progression. Although self-medication may appear cost-saving in the short term, it can ultimately increase healthcare costs due to treatment failure, complications, and the need for advanced care. The frequent use of antibiotics (38%) is particularly concerned in the context of antimicrobial resistance (AMR), a major global health threat. Evidence indicates that antibiotic self-medication is a key contributor to AMR worldwide, with systematic reviews reporting prevalence rates exceeding 60% among student populations [24]. Collectively, these findings suggest that self-medication among Somali university students mirrors global trends observed in low- and middle- income countries and contributes to the growing burden of AMR. Importantly, intervention studies have demonstrated that targeted educational programs can significantly improve students’ knowledge of AMR and promote safer attitudes and practices regarding antibiotic use.

This study makes an additional contribution to the existing body of evidence by being the first to assess self-medication practices among Somali undergraduates across multiple institutions. Our findings complement those from Afghanistan, where non-medical students demonstrated a high prevalence of antibiotic self-medication [25], and Saudi Arabia, where modifiable behavioral and socioeconomic factors were identified as key drivers of student self-medication [26]. By situating our results within this broader literature, the study provides context-specific evidence that not only highlights regional similarities but also underscores the unique challenges posed by weak pharmaceutical regulation and limited health infrastructure in Somalia.

This study is novel in that it is the first to examine self-medication practices among Somali undergraduates across multiple private universities. Beyond estimating prevalence, it provides new evidence on the influence of field of study, common symptoms, and weak pharmaceutical regulation as drivers of self-medication. These findings have direct practical relevance, underscoring the need for targeted educational interventions, improved awareness of antibiotic misuse risks, and stronger enforcement of prescription-only policies. By generating locally relevant evidence from a fragile health system, this study contributes to both the global literature and actionable public health strategies in Somalia.

### Limitation of the study

This study has several limitations that should be considered when interpreting the findings. First, cross-sectional design limits the ability to establish causal relationships between associated factors and self-medication practices. Second, reliance on self-reported data may have introduced recall or social desirability bias, potentially leading to under- or overestimation of behaviors. Third, the use of convenience sampling may limit the generalizability of findings beyond the included universities. These limitations are common in self-medication research and have been reported in similar studies conducted in Ethiopia and Saudi Arabia [19, 20]. Despite these limitations, the study provides novel and context-specific insights into self-medication practices among Somali undergraduates.

## Conclusion

This study demonstrates a high prevalence of self-medication among undergraduate students in the Banadir region of Somalia. Targeted educational interventions, particularly for non-medical students, are recommended to improve awareness of appropriate medicine use. Strengthening training on rational antibiotic use across university programs, alongside stricter enforcement of prescription-only regulations, may help reduce inappropriate self-medication practices. Despite limitations related to study design and self-reported data, this research provides the first multi-university evidence on self-medication among Somali undergraduates and offers valuable, context-specific contributions to the global literature on self-medication.

## Data Availability

The datasets generated and/or analysed during the current study are available from the corresponding author on reasonable request.

## Declarations

This study has been reported in accordance with the STROBE (Strengthening the Reporting of Observational Studies in Epidemiology) guidelines.

## Abbreviations

SM: Self medication
AOR: Adjusted odds ratio
CL: Confidence Interval
COR: Crude odds ratio
SPSS: Statistical Package for Social Sciences
IRB: Institutional Review Board
SMA: Self Medication Antibiotics
